# 

**Published:** 1860-01

**Authors:** 


### AMERICAN JOURNAL

OF

### DENTAL SCIENCE:

EDITED BY

CHAPIN A. HARRIS, M. D., D. D. S.

AND

A. SNOWDEN PIGGOT, M. D.

VOL. X—NEW SERIES.

Surgeon Gen'l's Office LIBRARY. 21645 Washington, D.C.

PHILADELPHIA:
LINDSAY & BLAKISTON.
LONDON:
TRUBNER & CO., 12 PATERNOSTER ROW.
1860.

W
1
AM 45 M

J. W. WOODS, PRINTER,

BALTIMORE.

*Our Contributors.*—We beg to remind our contributors of their recently made engagements. Surely that love for the diffusion of knowledge which, no doubt, animated them to promise communications has not exhausted itself in so short a time.

